# βArrestin-1 and Mcl-1 Modulate Self-Renewal Growth of Cancer Stem-Like Side-Population Cells in Non-Small Cell Lung Cancer

**DOI:** 10.1371/journal.pone.0055982

**Published:** 2013-02-13

**Authors:** Sandeep Singh, Namrata Bora-Singhal, Jodi Kroeger, Hanane Laklai, Srikumar P. Chellappan

**Affiliations:** 1 Department of Tumor Biology, H. Lee Moffitt Cancer Center and Research Institute, Tampa, Florida, United States of America; 2 Flow Cytomerty Core Facility, H. Lee Moffitt Cancer Center and Research Institute, Tampa, Florida, United States of America; University of South Alabama, United States of America

## Abstract

Side population (SP) cells have been reported to have properties of cancer stem-like cells (CSCs) in non-small cell lung carcinoma (NSCLC), yet their molecular features have not been fully elucidated. Here we show that, NSCLC-SP cells were enriched in G_0_/G­_1_ phase of cell cycle, had higher aldehyde dehydrogenase activity as well as higher clonogenic and self-renewing ability compared to main population (MP) cells. Interestingly, SP cells were also able to trans-differentiate into angiogenic tubules *in vitro* and were highly tumorigenic as compared to MP cells. SP-derived tumors demonstrated the intratumoral heterogeneity comprising of both SP and MP cells, suggesting the self-renewal and differentiation ability of SP cells are manifested *in vivo* as well. βArrestin-1 (βArr1) is involved in the progression of various cancers including NSCLCs and we find that depletion of βArr1 significantly blocked the SP phenotype; whereas depletion of βArr2 had relatively minor effects. Ectopic expression of βArr1 resulted in increased SP frequency and ABCG2 expression while abrogation of βArr1 expression suppressed the self-renewal growth and expansion of A549 cells. Anti-apoptotic protein Mcl-1 is known to be one of the key regulators of self-renewal of tissue stem cells and is thought to contribute to survival of NSCLC cells. Our experiments show that higher levels of Mcl-1 were expressed in SP cells compared to MP cells at both transcriptional and translational levels. In addition, Obatoclax, a pharmacological inhibitor of Mcl-1, could effectively prevent the self-renewal of both EGFR-inhibitor sensitive and resistant NSCLC cells. In conclusion, our findings suggest that βArr1 and Mcl-1 are involved in the self-renewal and expansion of NSCLC-CSCs and are potential targets for anti-cancer therapy.

## Introduction

Despite significant therapeutic advances, lung cancer causes the maximum number of cancer related deaths worldwide [1], [2]. According to the World Health Organization (WHO), lung cancer will cause about 2.5 million deaths per year by the year 2030 [3]. In the United States, approximately 85% of the patients diagnosed with NSCLCs, die of this disease within five years [4], [5]. These facts highlight a need for better understanding of the cellular and molecular events underlying the genesis of this disease for the development of more effective therapeutics. Cancer stem cell model has emerged as a viable explanation for the initiation and progression of the aggressive cancers like NSCLCs and are potential therapeutic targets [6], [7], [8], [9], [10].

Cancer stem cell model suggests that a subset of cells termed as cancer stem-like cells (CSCs) within the tumor have the deregulated properties of normal stem cells with sustained self-renewal, and can generate secondary tumors that recapitulate the heterogeneity and diversity of original tumor [9], [11], [12], [13], [14], [15]. Hoechst 33342 dye excluding cells, termed side-population (SP) cells, have been described to have CSC like properties in a variety of tumors, including NSCLCs [16], where they displayed increased tumorigenicity when transplanted into immunocompromised mice [17], [18] as compared to the main population (MP). SP phenotype is dependent on the differential ability of cells to efflux the Hoechst 33342 dye via the ATP-binding cassette (ABC) family of transporter proteins, mainly ABCG2 (also known as breast cancer resistance protein, BRCP1), which is specifically expressed on the cell membrane of stem cell populations [19]. Earlier studies have demonstrated the existence of SP cells in certain established human NSCLC cell lines [16] however, their detailed molecular characterization as well as functional ability to generate heterogeneous tumors remains to be elucidated. In this study, we provide comprehensive evidence that SP cells isolated from established human NSCLC cell lines and tumors are highly enriched with NSCLC-CSCs. In addition, we find that ALDH1, which has been identified as a marker for CSC from other types of tumors, are enriched in SP cells from NSCLC. Our molecular analyses show that stem cell like properties of SP cells is governed at least in part by the scaffolding protein, β-arrestin-1; in addition, the survival protein Mcl-1 plays a role in the self-renewal of these cells. Thus, it appears that targeting β-arrestin-1 or Mcl1 might be viable means of inhibiting the stem cell-like properties of SP cells from NSCLC.

## Materials and Methods

### Cell Lines and Reagents

The Non-small cell lung adenocarcinoma cell lines, A549, H1650, H460 and H1975 were obtained from ATCC and maintained in RPMI or DMEM containing10% fetal bovine serum (FBS; Mediatech) in 5% CO_2_ at 37°C. Although these cell lines were originally purchased from ATCC, we did not revalidate them. Fumitremorgin C (FTC) was purchased from Sigma Inc and Obatoclax was purchased from Selleck Chemicals LLC.

### RNA Preparation and Real Time qPCR Analysis

RNA extraction and cDNA preparation was followed as described earlier [20], [21], [22]. Real-time PCR was done with 1 µL of the cDNA in a MyiQ real-time PCR detection system (Bio-Rad) by using iQ SYBR Green PCR Supermix (Bio-Rad) according to the manufacturer’s guidelines. Fold inductions were calculated using the formula 2^–(ΔΔCt)^ using GAPDH as internal control gene. The gene-specific primer pairs were as follows. CD31 (F) 5′- CCTGACAGTGTCTTGAGTGGGTG -3′, CD31 (R) 5′- AGTGATTTTGGCTAGGCGTGGT -3′; βArr1 (F) 5′- AGAGTCTATGTGACGCTGACCTGC -3′, βArr1 (R) 5′- GTTCCTGCAGCCGCGTCAG -3′, Mcl-1 (F) 5′- ATGCTTCGGAAACTGGACAT -3′, Mcl-1 (R) 5′- TCCTGATGCCACCTTCTAGG -3′, GAPDH (F) 5′-GGT GGT CTC CTC TGA CTT CAA CA-3′, GAPDH (R) 5′-GTT GCT GTA GCC AAA TTC GTT GT-3′.

### Western Blot Analysis

Lysates from sorted SP and MP cells were prepared by NP40 lysis as described earlier [20]. In brief, sorted cells were washed twice with ice-cold PBS. The cells were spun at 800 *g* and lysed using M2 lysis buffer (20 mM Tris-HCl, pH 7.6, 0.5% NP-40, 250 mM NaCl, 3 mM EGTA, and 3 mM EDTA) containing protease inhibitors. Equal amounts of proteins (50 µg) were separated on SDS-PAGE and transferred to nitrocellulose membranes (Bio-Rad), blocked by 5% nonfat dry milk in PBS containing 0.1% Tween-20 and incubated with the appropriate primary antibodies. Monoclonal antibodies against ABCG2 and βArr1 were purchased from Millipore and polyclonal antibody against E2F1 and Mcl1 were purchased from Santa Cruz Biotechnology Inc.

### Hoechst 33342 Dye Efflux Assay for SP Analysis and Cell Sorting

Adherent cells were harvested using accutase reagent (Sigma Inc). For tumor explants, primary human tumor tissue grown in athymic nude mice was minced, enzymatically digested with 0.2% collagenase IV (Worthington Biochemical Corporation) prepared in 10% FBS containing medium for 60 minutes at 37°C. The digest was further disaggregated by passing through 10 ml pipette several times and filtered through 100/70-µm cell strainer to obtain a single cell suspension. Cells were washed and resuspended in DMEM/F12K with 2% FBS at 1×10^6^ cells/ml density and incubated with 4 µg/ml of Hoechst 33342 dye (Invitrogen) for 90 minutes at 37°C in presence or absence of 1 µM FTC, as described by Goodell et al. [23]. Cells were incubated with 2 µg/ml Propidium iodide (PI; Sigma Inc) before analysis to visualize and exclude the non-viable cells. The Hoechst 33342 dye was excited at 350 nm using UV laser and its fluorescence was analyzed using 400–500 nm BP filter for blue emission and 640–680 nm BP filter in combination with 655 nm LP-filter for red emission. Flow cytometers from BD Biosciences were used for data acquisition. Data were acquired using LSRII or FACS Vantage (DiVa), and sorted using FACS Vantage (DiVa) cell sorter. Data analyses were done using FlowJo software (Tree Star).

### Aldefluor Assay

The Aldefluor kit was used to profile and separate cells with high Aldh activity as per the instruction of the manufacturer (Stem Cell Technologies). Briefly, Hoechst 33342 stained cells were incubated with Aldh protein substrate BODIPY-aminoacetaldehyde (BAAA) in Aldefluor assay buffer supplemented with 1 µM FTC to prevent the efflux of Hoechst dye. After 45 minutes of incubation at 37°C, cells that could convert BAAA to its fluorescent product BODIPY-aminoacetate (BAA) were considered Aldh-Hi. The gates for flow cytometric analysis were drawn relative to baseline fluorescence, which was determined by the addition of Aldh-specific inhibitor diethylaminobenzaldehyde (DEAB). Samples treated with DEAB alone served as negative control.

### Sphere Formation or Self-renewal Assay

Sorted SP or MP cells were plated in ultra-low adhesion 96 well plates (Corning) at the density of 10,000 cells/ml (1000 cells/well in 100 µl medium) in serum free DMEM/F12K (1∶1) (Invitrogen), supplemented with 1X-N2 supplement (Invitrogen), 10 ng/ml EGF and 10 ng/ml bFGF (Sigma) and allowed to grow for 10 days. Images of the spheres were taken using phase contrast microscope (Nikon) and total number of spheres more that 60 µm were counted. To study the effect of drugs on the self-renewal of SP cells, drugs were added to the respective wells on day 1 and 5 and size (>60 µm) and number of the spheres were analyzed on day 10.

#### *In vivo* tumor formation assay

5-weeks-old female SCID-beige mice were used for these experiments under a protocol that was approved by the Institutional Animal Use and Care Committee at the University of South Florida (Animal Welfare Assurance Number A4100-01). Sorted SP or MP cells from H1650 cell line were washed with serum-free DMEM-F12K and suspended in a 1∶1 mixture of growth factor reduced Matrigel (BD) and HBSS at indicated numbers and implanted subcutaneously. Tumor volumes were determined once a week by measuring length (*L*) and width (*W*), and volume calculated using the formula *V = *1/2×*L*×*W* ^2^as described earlier [21], [22], [24], [25]. The the health status of mice was monitored daily for signs of discomfort, including inactivity, hypoactivity, hyperactivity, restlessness, self-trauma, aggressiveness, isolation from cage mates, ataxia, shallow, rapid and/or labored breathing, pale mucous membranes, cyanosis, failure to groom, cachexia, soiled anogenital area, failure to respond to stimuli, lack of inquisitiveness, vocalization, ascites, and/or hunched posture. All efforts were made to minimize suffering. At the end of the experiment, the animals will be euthanatized by exposure to increasing concentrations of CO_2_ in a dedicated CO_2_ chamber; compressed gas was used a source for CO_2_.

### Statistical Methods

Data were presented as the mean ± standard deviation (SD). To assess the statistical significance of differences, student’s *t* test was performed. All the statistical analyses were performed using the Microsoft Excel software. The data were considered statistically significant when the *p* value was less than 0.05.

## Results

### SP Cells from NSCLC Demonstrate Stem Cell-like Properties

**Figure 1:**
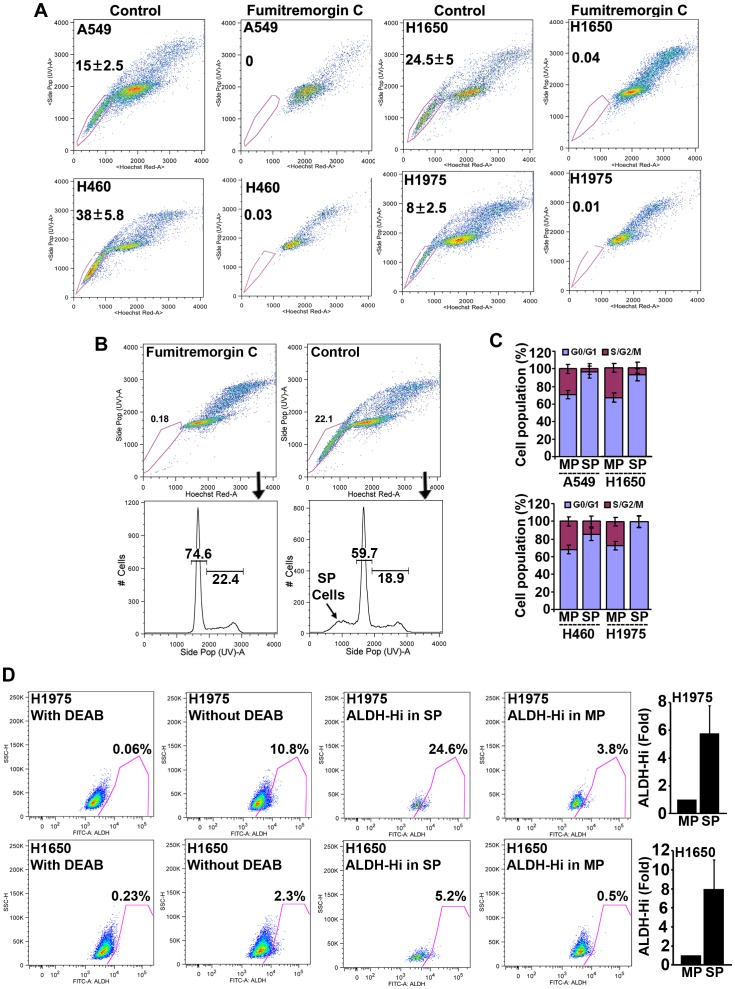
Characterization of SP cells by flow cytometry. (**A**) FACS analysis on single cell suspension of human NSCLC cell lines stained with Hoechst 33342 dye showing SP cells. SP cells are enclosed within the area demarcated in pink. Fumitremorgin C inhibited the efflux of the dye and caused the disappearance of SP cells. The frequency of SP cells is represented. (**B, C**) Cell cycle analysis using area-histogram parameter for the blue emission of Hoechst 33342 as described in the text. The profile shown in B is the representative for all the four cell lines. (**D**) Aldefluor assay on Hoechst 33342 stained H1975 and H1650 cells. The base line fluorescence was established by inhibiting ALDH activity with DEAB (Left) to generate the gate to identify ALDH-Hi cells in total as well as the SP and MP cells that have not been incubated with DEAB (three right panels). All ALDH-Hi cells are enclosed within the area demarcated in pink. The fold difference in frequency of ALDH-Hi cells between SP and MP cells is plotted.

Attempts were made to assess the percentage of SP cells in four different human NSCLC cell lines, chosen on the basis of K-Ras or EGFR mutation status. A549 (K-Ras mutant; wild type EGFR amplification) and H460 (K-Ras mutant) as well as H1650 (EGFR mutant; Exon 20 deletion: delE746-A750) and H1975 (EGFR mutant; L858R and T790M mutations) cells contained SP-cells with varying frequency ranging from approximately 8% (H1975) to 40% (H460). A specific inhibitor of ABCG2 [26], Fumitremorgin C (FTC) could block the appearance of SP cells (Figure 1A), suggesting that this particular transporter plays a specific role in maintaining the SP phenotype. These results suggest that SP cells are present in NSCLC lines, and their prevalence is independent of K-Ras or EGFR mutation status.

It has been proposed that normal tissue stem cells and stem-like tumor initiating cells have different cell cycle profiles compared to differentiated cells, and they progress through the cell cycle slower [27], [28]. Given this background, we made attempts to examine whether the cell cycle profile of SP cells differed from that of the differentiated MP cells. To examine the cell cycle distribution, the histogram profile generated from the area parameter for blue emission of Hoechst 33342 was used to examine the cell cycle phase distribution for SP and MP cells. Since FTC treatment allows both SP and MP cells to retain the Hoechst 33342 dye, this sample was used to mark the boundaries for G_1_ and S-G_2_/M phases, based on their DNA content. Similar gates were applied for the samples where FTC was not added and thus SP cells appeared as a sub-G_0_/G_1_ population (Figure 1B). Upon comparison of the distribution of FTC treated and untreated cells, it was found that 80–100% of SP cells were in the G_0_/G_1_ phase of the cell cycle, dependent on the cell line (Figure 1C). In all cases, SP cells had more G_0_/G_1_ cells than MP cells from the same cell line, suggesting that they have attenuated cell cycle progression characteristic of stem-like cells.

Experiments were conducted to examine whether other cancer stem cell markers are expressed on SP cells. Towards this purpose, flow cytometry was performed for the activity of aldehyde dehydrogenase (Aldh-Hi) which has been recently used as a potential marker to identify CSCs in NSCLCs [29], [30]. Analysis using the Aldefluor assay kit on H1650 and H1975 cell lines showed 8 and 6 fold higher frequency of Aldh-Hi cells in SP cells as compared to MP cells, suggesting that SP cells isolated from NSCLC cell lines are enriched in stem like cells (Figure 1D), as seen by the expression of a different marker. Overall, approximately 1.3% of H1650-SP cells and 2% of H1975-SP cells were Aldh-Hi cells.

### SP Cells Display Stem Cell-like Characteristics *in vitro*

**Figure 2:**
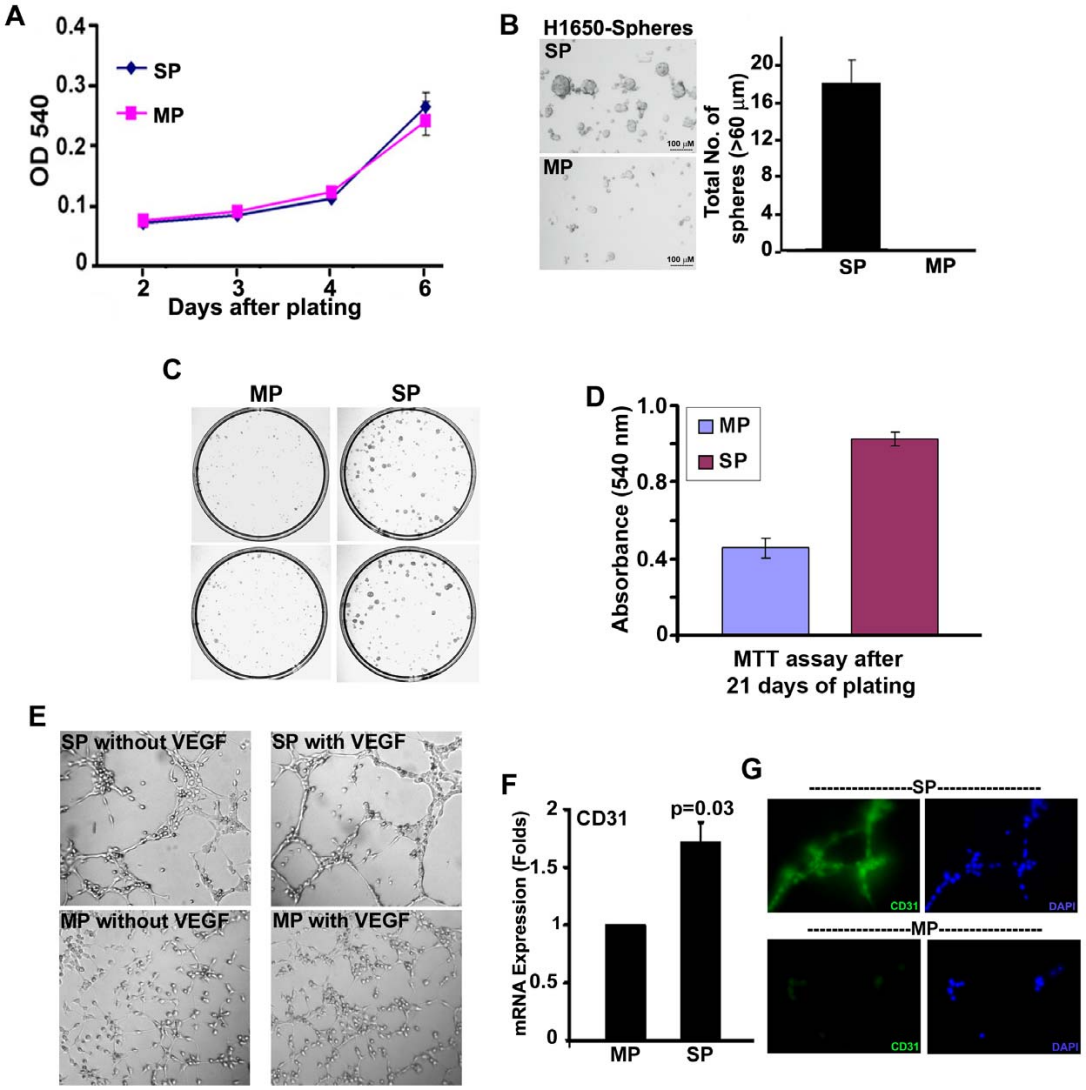
NSCLC-SP cells have cancer stem cell-like properties *in vitro*. (**A**) Growth curve on 10,000 H1650-SP or MP cells were generated using MTT cell proliferation assay in regular growth medium [22]. (**B**) SP or MP cells from H1650 cell line were plated in serum free medium supplemented with EGF and bFGF for 10 days. The average (±SD) number of spheres from 1000 cells is plotted. (**C**) H1650-SP cells resulted in larger and more colonies compared to MP cells when plated at the density of 1000 cells per 60 mm plate. (**D**) Cell viability was assayed after 21 days of plating via MTT assay. (**E**) SP or MP cells were plated on Matrigel and grown in endothelial growth medium (Lonza) overnight. SP cells gave rise to angiogenic like tubules effectively. (**F**) Real time qPCR analysis on SP and MP cells performed for *CD31* mRNA expression. (**G**) CD31 expression on angiogenic tubules as visualized by immunofluorescence.

*In vitro* experiments were conducted to further characterize the SP and MP cells. Upon cell sorting, cells were plated at high density (10,000 cells/well in 96 well plate) in complete media and monitored for 6 days by MTT assay. H1650-SP and MP cells had comparable proliferation capacity when grown in complete media under adherent conditions (Figure 2A), suggesting that both the sorted cell fractions are equally healthy and Hoechst 33342 dye staining for SP analysis and cell sorting protocol had no deleterious effect on these cells. One characteristic feature of stem cells is their ability to self-renew and also to give rise to more differentiated cells, through asymmetric division [15]. Self-renewing normal or cancer-stem like cells can grow as non-adherent spheres when cultured at low density in serum free, stem cell selective medium; differentiated cells do not grow or form the spheres in this medium [13], [31], [32]. The self-renewal property of SP cells was examined by performing sphere formation assay using SP and MP cells isolated from H1650 cells. While SP cells were able to grow as spheres, MP cells had markedly less capacity to grow under similar conditions (Figure 2B) suggesting that self-renewal ability characteristic of stem cells is displayed only by SP cells. We then examined the clonogenic potential of both SP and MP cells by plating the cells and culturing at clonal density (1000 cell in 60 mm plate) for 21 days in FBS containing media. In this panel, cells are plated at very low density, and their long term proliferation and colony forming ability was checked. As shown in Figure 2C, formation of large colonies was substantially greater among SP cells than it was among MP cells. The viability of the colony forming cells was determined by MTT assay (Figure 2D), collectively providing evidence that SP cells display increased clonogenicity.

Certain types of tumors have been reported to demonstrate vascular mimicry, due to the ability of tumor cells to acquire properties of endothelial cells and other cellular components of the vasculature [33], [34], [35]. The role of cancer stem cells in this context has been examined and, recent evidences suggest that CSCs from gliomas could trans-differentiate into endothelial lineage [33], [34], [35]. Given this background, we examined the capacity of H1650 SP cells to form angiogenic tubules *in vitro*. H1650-SP cells plated on matrigel formed a network of angiogenic tubules upon treatment with VEGF, whereas MP cells were significantly impaired in this ability (Figure 2E). It was found that MP cells remained relatively unorganized, compared to the organized tubule like structures formed by the SP cells. Experiments were conducted to assess whether the tubule like structures formed by SP cells display biochemical features of angiogenic vessels. Towards this purpose, a real-time PCR analysis was first conducted, which showed a higher expression of endothelial specific *CD31* mRNA in SP cells as compared to MP cells (Figure 2F). Further, the immunofluorescence staining of the tubules showed that these tubules formed by SP cells were strongly positive for CD31 expression (Figure 2G), suggesting that SP cells can attain an endothelial lineage. These experiments reveled that SP cells isolated from a NSCLC cell line can transdifferentiate into endothelial-like cells and might be able to give rise to angiogenic tubules in the tumors.

### SP Cells are Enriched with Tumorigenic Cells and Demonstrated Self-renewal and Differentiation of SP Cells *in vivo*

**Figure 3:**
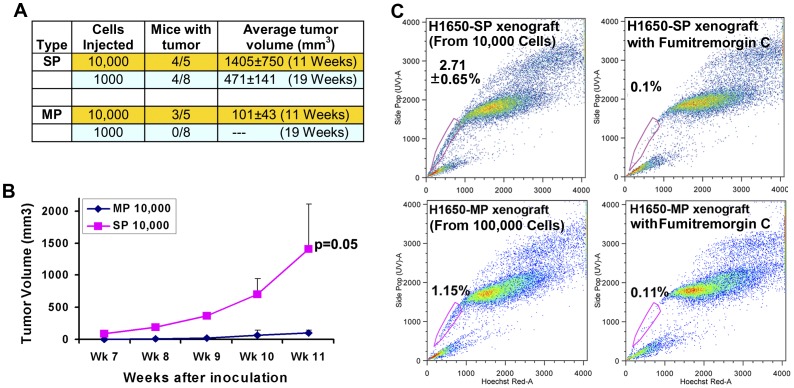
SP cells from NSCLC cell lines have cancer stem cell-like properties *in vivo*. (**A**) Sorted SP and MP cells from H1650 cell line were implanted into the flanks of SCID mice. Tumor incidence and the average volume (±standard error) of the tumors formed within the indicated time are tabulated. (**B**) Growth kinetics of tumors from SP and MP cells, measured weekly. The points represent the average (±standard error). (**C**) Subcutaneous tumors derived from 10,000 H1650-SP cells or 100,000 H1650-MP cells were resected and analyzed for SP phenotype in the presence or absence of FTC. Tumors from SP and MP cells contained predominantly differentiated MP cells.

Given the ability of SP cells to self renew and differentiate into angiogenic tubules as well as MP cells, we next examined the ability of SP or MP cells to form tumors in SCID mice. SP and MP cells from H1650 cell line were implanted subcutaneously on the dorsal flanks of SCID mice and tumor growth assessed weekly by caliper measurements. As shown in Figure 3A, four mice (n = 5) injected with 10,000 SP cells produced large tumors (volume ∼1400 mm^3^) within 11 weeks of implantation. However, similar numbers of MP cells were significantly impaired in their ability to form tumors within the same time frame; the tumors were relatively smaller and remained close to the implanted size (volume∼100 mm^3^) (Figure 3B). Further, SP cells formed tumors even with 1,000 cells (volume ∼471±141 mm^3^) after 19 weeks of implantation, whereas similar number of MP cells were failed to generate any tumors (Figure 3A).

To elucidate whether tumors generated from SP cells had heterogeneous SP and MP cells, Hoechst 33342 staining and SP analysis was performed after enzymatic dissociation of subcutaneous tumor xenografts. Tumors generated from 10,000 H1650-SP cells were mainly composed of MP cells whereas SP cells were maintained at approximately 3% frequency within the tumor (Figure 3C, upper panel). These results indicate that SP cells are highly enriched with CSCs that are able give rise to tumors as well as self-renew and differentiate themselves *in vivo*. However, tumors generated in one mouse (out of three) upon implantation of 100,000 H1650-MP cells demonstrated the dedifferentiation of MP cells to generate SP cells *in vivo* (Figure 3C, lower panel), after 9 weeks in these mice (Figure 3B). Indeed, it has been reported that differentiated cancer cells might be able to de-differentiate and acquire stem-like properties in certain cases [36], probably facilitating the delayed growth of tumors.

### βArr1 Regulates the Self-renewal Growth of SP Cells

**Figure 4:**
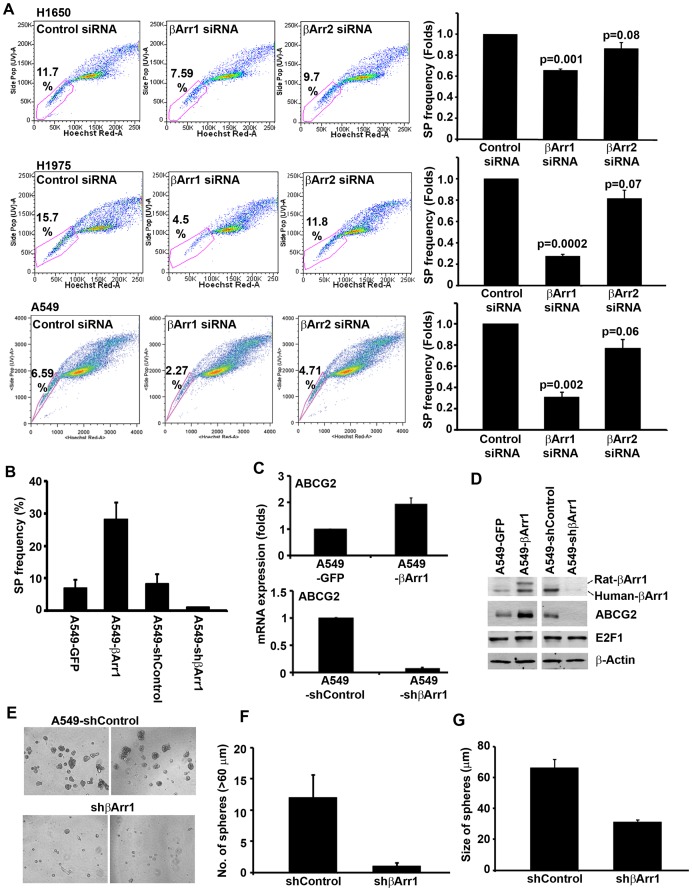
βArr1 regulates the self-renewal of SP cell. (**A**) H1650, H1975 and A549 cells were transfected with siRNA against βArr1 and βArr2. Frequency of SP cells was compared with non-targeting control siRNA transfected cells. SP cells are enclosed within the area demarcated in pink. Bar diagrams represent the fold difference in frequency in siRNA transfected cells in respective cell lines. (**B**) SP frequency was analyzed and represented for A549 cells ectopically expressing rat-βArr1 or GFP and shRNA against βArr1 or non-targeting control shRNA. (**C**) Real-time PCR and (**D**) western-blot analysis for ABCG2 expression was performed for these stable cell lines. (**E, F, G**) A549 cells stably expressing shRNA against βArr1 and non-targeting shRNA were plated for self-renewal assay. (**E**) Phase contrast microscopy images of the spheres in presence or absence of drugs are presented. (**F, G**) Average number and size of the spheres generated per well from 1000 cells is plotted (mean±SD).

βarrestin-1 (βArr1) is a scaffolding protein that plays a major role in the desensitization of G-protein coupled receptors [20], [37], [38], [39], [40]. Recent studies have shown that βArr1 plays a role in the activation of Src kinases by various receptors and might facilitate the proliferation of lung cancer cells [38], [41]. Further, βArr1 levels were found to be elevated in metastatic NSCLC and other cancers compared to normal tissue or primary tumors [20], [39], [40], [41]. Given the correlation of βArr1 with tumor progression and metastasis, we examined whether this protein plays a role in the stem cell-like properties of SP cells. In the first set of experiments, siRNAs to βArr1 or the related βArr2 genes were transfected into H1650, H1975 and A549 cells; a non-targeting siRNA was used as the control. It was found that transient transfection of βArr1 specific siRNA decreased the frequency of SP cells by approximately 40 to 70%; the reduction was only 10 to 20% when βArr2 siRNA was transfected (Figure 4A); suggesting an important role of βArr1 in regulation of SP frequency. To further elucidate the role of βArr1 in the stem-like functions of SP cells, we generated A549 cells that stably overexpressed rat βArr1, or those that were stably transfected with a shRNA to βArr1. It was found that cells stably overexpressing βArr1 had approximately three fold more SP cells compared to control A549 cells that stably expressed GFP (A549-GFP). Conversely, stable depletion of βArr1 using a specific shRNA (A549-shβArr1) resulted in a significant decrease in SP frequency as compared to a control cell line that was stably transfected with a non-targeting shRNA (A549-Control) (Figure 4B). Real-time PCR experiments were conducted to assess whether the changes in SP frequency correlated with different levels of ABCG2. It was found that A549 stably overexpressing βArr1 had two fold more ABCG2 message compared to cells transfected with GFP. Conversely, cells depleted of βArr1 had significantly lower ABCG2 message compared to control shRNA expressing cells (Figure 4C). These results were confirmed by western blotting (Figure 4D); it was found that βArr1 null cells had lower amounts of ABCG2 as well as βArr1, but comparable amounts of β-actin and E2F1, which were used as controls.

Given the role of βArr1 in generation of SP cells, we next examined whether this protein facilitates self-renewal as well. Towards this purpose, SP cells were isolated from A549 cells stably expressing a shRNA against βArr1 or a non-targeting control shRNA and self-renewal property assessed by sphere formation assays. It was found that cells lacking βArr1 had markedly lower number of spheres (Figure 4E); further, the size of the spheres formed by βArr1 null cells was markedly lower than those formed by SP cells from control cells (Figure 4F, G). These experiments clearly show that the scaffolding protein βArr1 plays a role in the establishment of SP cells through the regulation of ABCG2 expression and also facilitates the self-renewal property of these NSCLC stem-like cells.

### Inhibitor of Mcl-1 Targets the Self-renewal Growth of SP Cells

**Figure 5:**
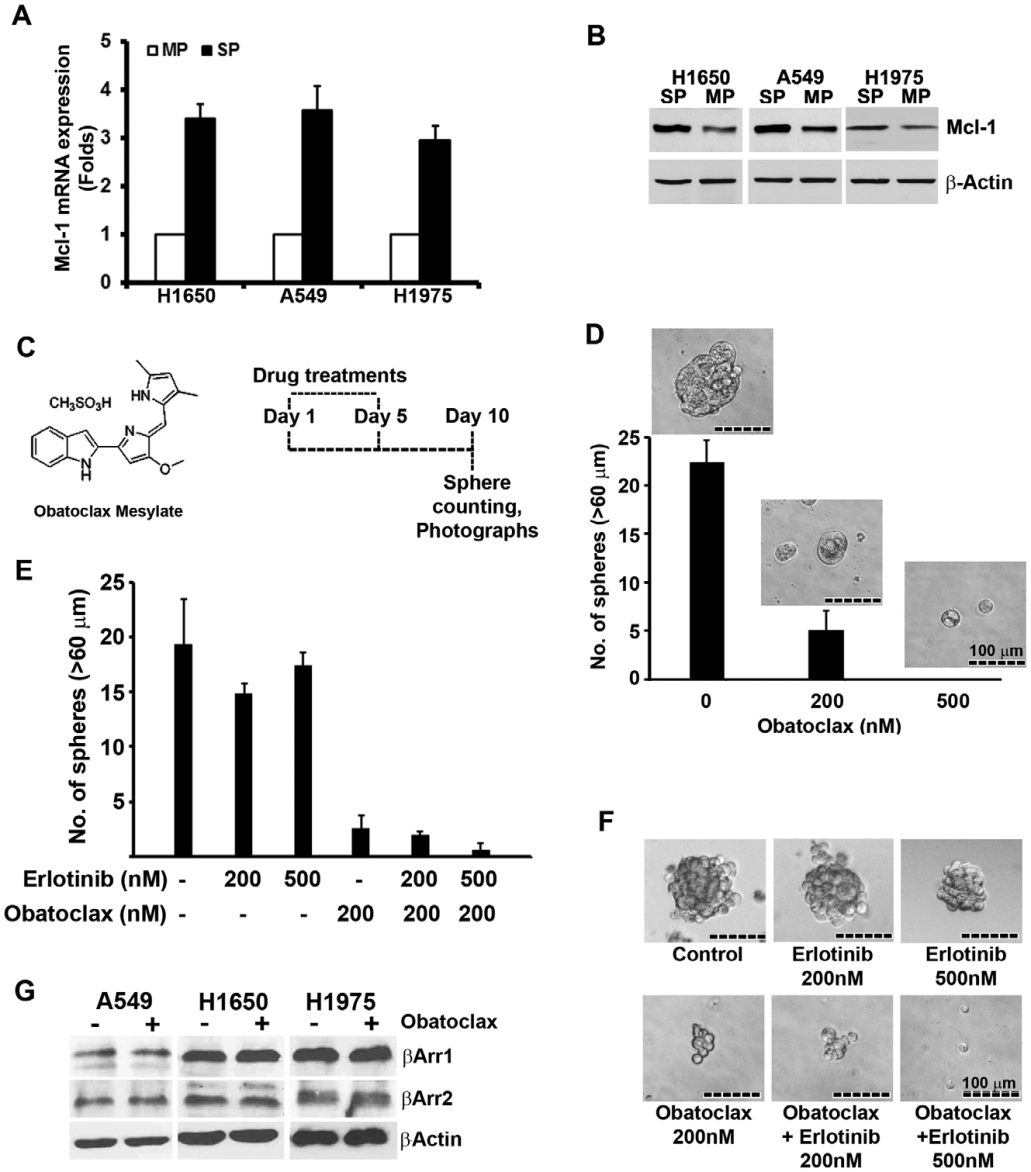
Mcl-1 regulates the self-renewal of SP cell. (**A, B**) Relative expression of Mcl-1 was examined at mRNA and protein levels for indicated cell lines by RT-PCR and western blot analysis. (**C**) Structure of Obataoclax and its treatment schedule is represented. (**D**) H1650-SP cells were sorted and plated for self-renewal assay in the presence or absence of Obatoclax at indicated concentration. Average number of spheres generated per well from 1000 cells is plotted (mean±SD). Phase contrast microscopy images of the spheres in presence or absence of drugs are presented. (**E**) SP cells were sorted from erlotinib resistant H1975 cell line and plated for self-renewal assay in the presence or absence of indicated drugs. Average number of spheres generated per well from 1000 cells is plotted (mean±SD) and (**F**) phase contrast microscopy images of the spheres in presence or absence of drugs are presented. (**G**) Western-blot analysis of βArr1 and βArr2 in Obatoclax treated cells. Inhibition of Mcl-1 did not affect the expression of βArr1 and βArr2 in any of the cell lines tested.

Enhanced cell survival and drug resistance is a feature of cancer stem cells [10], [42]. The anti-apoptotic protein Mcl-1, is among the most frequently amplified genes in human cancer including NSCLCs [22], [43], [44], . Recently, studies have also associated the Mcl-1 function with regulation of self-renewal of hematopoietic stem cells [48], [49]. Given the potential role of Mcl-1 in hematopoietic stem cells, we next explored whether Mcl-1 function contributes to the self-renewal property of NSCLC-SP cells. As a first step, qRT-PCR was conducted on RNA isolated from SP and MP cells isolated from H1650, A549 and H1975 cell lines. Mcl-1 message was expressed at higher levels in SP cells from all the three cell lines (Figure 5A). The Mcl-1 protein level was also elevated in SP cells compared to MP cells in all the three cell lines, as seen by western blotting (Figure 5B).

Obatoclax is a hydrophobic molecule (Figure 5C) that was developed as a Bcl-2 family antagonist including Mcl-1 [50]. Obatoclax has been shown to overcome resistance to apoptosis mediated specifically by Mcl-1 [51]. Therefore, here we used Obatoclax to explore the role of Mcl-1 in self-renewal of SP cells, by conducting sphere formation assays in the presence or absence of Obatoclax. As shown in Figure 5C, cells were seeded at day 1 for sphere formation assay and Obatoclax was added on day1 and day 5 at indicated dose. After 10 days of plating the number of spheres were counted and analyzed. As shown in Figure 5D, inhibition of Mcl-1 activity by 200 nM of Obatoclax demonstrated a 4–5 fold decrease in the number of spheres; further the size of the spheres was also significantly reduced as shown in the picture above the bar (Figure 5D). 500 nM concentration of Obatoclax completely suppressed the sphere formation (Figure 5D).

Erlotinib and gefitinib are inhibitors of EGFR that show significant efficacy in treating NSCLC patients harboring EGFR mutations [52], [53]. At the same time, a secondary point mutation in exon 20 of EGFR (T790M) is associated with acquired resistance to EGFR inhibitors, including Erlotinib [52]. We tested the effect of 200 nM of Obatoclax on self-renewal growth of SP cells from H1975 cell line, which harbors Erlotinib-resistant-T790M mutation along with Erlotinib responsive-L858R mutation in exon 21 [54]. As expected, self-renewal of H1975-SP cells were resistant to Erlotinib treatment, but at the same time, Obatoclax alone or in combination to Erlotinib could significantly inhibit the self-renewal of these cells (Figure 5E and F). Overall, these results suggested that Mcl-1 activity contributes to maintenance of the self-renewal of SP cells and this could be targeted by the anti-Mcl-1 drug Obatoclax.

Since βArr1 was found to regulate the SP frequency and self-renewal of these stem-like cells, we next attempted to explore if Obatoclax treatment may result in attenuating the levels of βArr in NSCLC-cells. However, the levels of both βArr1 and βArr2 were not altered all the three NSCLC-cell lines treated with 500 nM concentration of obatoclax under similar conditions which has resulted in decreased self-renewal growth of these cells (Figure 5G). We next checked if βArr expression may have direct correlation with Mcl-1 expression in these cells. βArr 1 and βArr2 levels were depleted through siRNA in three different NSCLC-cell lines and levels of Mcl-1 was detected by RT-PCR analysis. Data suggested that Mcl-1 expression was not dependent on the βArr levels in these cells (Figure S1). Therefore, these results suggest that βArr1 and Mcl-1 might be regulating stemness through different pathways.

## Discussion

The cancer stem cell model of tumor initiation and growth suggests that the tumors are maintained by a subpopulation of stem or progenitor-like cells [8]. Deregulated self-renewal of cancer stem cells is proposed as one of the mechanisms to fuel the uncontrolled growth of cancer [9], [11], [12], [13], [14], [15]. Therefore, identification of the key regulators of self-renewal of cancer stem cells for specific tumors has become an appealing strategy for finding the suitable target for treatment [42]. In the current study, we used the SP phenotype to identify, enrich and characterize a subpopulation of NSCLCs with the properties ascribed to CSCs. Also, we demonstrate the specific and significant roles for βArr1 and Mcl1 gene functions in facilitating the self-renewal of the side population cells from NSCLCs.

Our study confirmed the presence of SP cells irrespective of the K-Ras or EGFR mutation status in established human NSCLC cell lines with the properties of CSCs as suggested by certain earlier studies [16], [55]. ALDH is a family of intracellular enzymes that participates in cellular detoxification, differentiation and drug resistance in stem cells [56]. In addition to the SP phenotype, another method for identifying and selecting stem cell population based on functional property is the specific high-ALDH activity of stem cells. ALDH activity is found to directly regulate the self-renewal of hematopoietic stem cells by inhibiting the endogenous retinoic acid biosynthesis [57]. High ALDH activity has recently been identified as a promising cancer stem cell marker for NSCLCs [29], [30]. Whereas, there was no significant difference in the expression of CD24, CD44 and CD133 between SP and MP cells (data not shown), we found that SP cells are enriched with ALDH-Hi cells by approximately 6–8 folds. Comparing the percentage of Aldh-Hi cells as well as the sphere forming cells in SP cells, we estimate that approximately 1–2% of SP cells from established cell lines may have stem-like properties. Cell cycle analysis suggest that the SP cells are relatively slow growing with almost 80–100% of the cells were found to be in G_0_/G_1_ phase of cell cycle, as suggested for CSCs [28]. In an earlier study, DNA replication associated protein MCM7 is shown to be expressed at lower levels as compared to its expression in MP cells [16]. This observation further supports the slow cell cycle progression of NSCLC-SP cells.

Within the tumors the CSCs may be represented by the restricted progenitor cells too [58]. For lung tumorigenesis, this proposal can be supported by a recent report where Sca-1-positive-BASCs were originally proposed as cell of origin for K-Ras (G12D) driven bronchioalveolar carcinoma [59], however, within the tumors, both Sca-1-positive as well as negative cells acquired cancer stem cells properties as demonstrated by their ability to initiate secondary tumors when implanted in recipient mice [60]. Therefore, CSCs may show more distinct markers than the proposed or studied so far, which represents the major challenge in identification and isolation of the CSCs including for NSCLC tumors [61]. As we have performed in our present study, characterization of putative CSCs based on the combination of functional properties like dye efflux, high-ADLH activity and slow cell cycle progression may represent an alternative approach for isolation of lung CSCs.

In the present study, we have provided several evidences supporting that the SP cells are enriched in NSCLC-CSCs. SP cells were found to be more tumorigenic *in vivo*, confirming the enrichment of tumor initiating cells in SP compartment. The data further showed that SP cells could give rise to a heterogeneous lung tumor *in vivo*. Tumors generated by pure SP cells were composed of both SP and MP cells, resembling the unsorted population, and thus showing the properties of self-renewal and differentiation of SP cells. Importantly, some of the MP cells implanted mice also demonstrated the latent-tumor initiation. The SP analysis of MP-cells-generated tumors suggested that some of the MP cells may also get de-differentiated into SP cells and acquire the properties of CSCs. As observed by us (data not shown) and by others, slow generation of SP cells from MP cells are reported earlier in *in vitro* culture assays too [16]. NSCLC-SP cells were capable of extensive proliferation as revealed by clonogenic ability and self-renewal potential as demonstrated by sphere formation assay. Further, we find that SP cells isolated from H1650 cell line demonstrate endothelial cell properties and *in vitro* trans-differentiation into vasculogenic cells on matrigel, as reported for glioma CSCs [33], [34], [35]. Together, with the tumorigenic ability with generation of heterogeneous tumor along with the exhibition of extensive proliferation and self-renewal phenotype and trans-differentiation capability, collectively suggest that SP cells isolated from NSCLC cell lines have stem-like properties.

Understanding the molecular mechanisms governing the deregulated self-renewal of SP cells may identify novel therapeutic targets to combat NCSLCs. Interestingly, βArr1 appears to regulate cell invasion and metastasis upon exposure to extracellular stimuli in various cancer models including lung cancer [37], [38], [39], [40]. βArr1 is also shown to play a major role in the metastasis of colorectal cancer [62]. In a recent report, βArr1 was significantly elevated in acute lymphoblastic leukemia patients [63]. βArr–GPCR complexes were found to be key players in ERK activation [64]. In addition, recently, our laboratory has demonstrated the functional role of βArr1 in nicotine induced cell proliferation, invasion and metastasis of NSCLCs [20]. Our data showed that βArr1 is required for nAChR-mediated activation of the MEK/ERK pathway in NSCLCs in Src dependent manner [20], [41]. βArr1-Src signaling axis is also reported in mediating prostaglandin induced signaling in lung cancer cells [38]. In ovarian cancer cells, βArr1 is linked with the endothelin-1-induced activation of Akt, GSK-3β in integrin-linked kinase (ILK) activation [37]. These results suggest that βArr1 is a multifunctional scaffold protein mediating many intracellular signaling networks in various tumor types. Our current observation that βArr1 signaling affects stem-like functions of SP cells is fascinating. Further demonstration of the molecular mechanism may suggest a novel βArr1 mediated signaling network to target against NSCLCs-CSCs.

Likewise, we also demonstrate Mcl-1 as another possible target against the self-renewal growth of NSCLC-CSCs. Recent studies have suggested the detrimental role Mcl-1 in maintenance of self-renewal and differentiation of hematopoietic stem cells of mouse and human origin. Among several Bcl-2 family members, only Mcl-1 was found to be up-regulated exclusively in the human hematopoietic stem cells and found to be indispensible for self-renewal of stem cells *in vivo* [48]. Similarly, Mcl-1 function is correlated with survival of early hematopoietic progenitor cells in mouse bone marrow [49]. Various studies have shown the higher expression of Mcl-1 in several cancers including NSCLCs and associated with resistance to the available treatments [22], [43], [44], [45], [46], [47]. Among NSCLCs, targeting Mcl-1 expression is suggested to be a viable approach against a subset of the disease [45], [46], [47]. Here, in the present study we showed the higher expression of Mcl-1 in NSCLC-SP cells as compared to its more differentiated counterpart; MP cells. Further, inhibition of Mcl-1 function by Obatoclax could effectively block the self-renewal of SP cells, even in the cell type that is resistant to the inhibitor of EGFR tyrosine kinase, Erlotinib. Overall, our results for the first time suggest that the inhibition Mcl-1 may effectively target the CSCs of NSCLC origin. Data also suggested that although both βArr-1 and Mcl-1 regulate the self-renewal growth of NSCLC SP cells but they may not be dependent on each-others for their expression and thus may regulate the self-renewal of SP cells through independent mechanisms.

In conclusion, the present study has demonstrated that SP cells are enriched with slow-cycling, ALDH-positive cells. SP cells were highly tumorigenic with self-renewal, differentiation as well as trans-differentiation ability. These characteristics make SP cells as critical therapeutic target against NSCLCs. Our findings suggest that βArr1 and Mcl-1 are the novel targets against the self-renewal growth of SP cells. Further mechanistic elucidation of these observations and *in vivo* validation will aid in the effective implementation of this study against NSCLCs progression.

## Supporting Information

Figure S1 **βArr1 does not regulate the Mcl-1 expression in NSCLCs. (A and B)** H1650 and H1975 cells were transfected with siRNA against βArr1 and βArr2 and real-time PCR for Mcl-1 expression was performed. Control siRNA was used as a negative control for transfection. Bar diagrams represents the average fold change in expression of the indicated genes in siRNA transfected cells in H1650 and H1975 cell lines.
(DOCX)
