# Supplementary material for: βArrestin-1 and Mcl-1 Modulate Self-Renewal Growth of Cancer Stem-Like Side-Population Cells in Non-Small Cell Lung Cancer
Source: PLoS One. 2013 Feb 13;8(2):e55982. doi: 10.1371/journal.pone.0055982 (PMC3572139; doi:10.1371/journal.pone.0055982)
Supplement: Figure S1 — βArr1 does not regulate the Mcl-1 expression in NSCLCs. (A and B) H1650 and H1975 cells were transfected with siRNA against βArr1 and βArr2 and real-time PCR for Mcl-1 expression was performed. Control siRNA was used as a negative control for transfection. Bar diagrams represents the average fold change in expression of the indicated genes in siRNA transfected cells in H1650 and H1975 cell lines. (DOCX) [file pone.0055982.s001.docx]

**

**

**Figure S1.**
